# FML/QuilA-Vaccinated Dogs Naturally Infected with *Leishmania infantum*: Serum Cytokines, Clinicopathological Profile, and Parasitological Parameters

**DOI:** 10.1155/2021/3192960

**Published:** 2021-10-05

**Authors:** Gregório Guilherme Almeida, Fernanda Morcatti Coura, Jonata de Melo Barbieri, Ana Carolina Junqueira Moura, Fabiola de Oliveira Paes-Leme, Adriane Pimenta da Costa-Val

**Affiliations:** ^1^Departamento de Patologia Geral, Universidade Federal de Minas Gerais, Belo Horizonte, Minas Gerais, Brazil; ^2^Instituto Federal de Educação, Ciência e Tecnologia de Minas Gerais, Campus Bambuí, Minas Gerais, Brazil; ^3^Escola de Veterinária, Universidade Federal de Minas Gerais, Belo Horizonte, Minas Gerais, Brazil

## Abstract

Dogs are the main reservoir of *Leishmania infantum* in endemic regions. Canine leishmaniasis, caused by *L. infantum*, can progress to a chronic disease resulting in death. Vaccines have been developed with a certain degree of success. The pathogenesis of this disease is not completely understood, especially in previously vaccinated dogs. We herein described clinical data, parasite load, serum levels of cytokines, and the reservoir potential in vdogs vaccinated with the fucose-mannose ligand (FML)/QuilA saponin vaccine (Leishmune™) naturally infected (Vi) and compared to vaccinated not infected dogs (Vn). Thirty-four dogs from private owners were divided into two groups: vaccinated/infected and vaccinated/uninfected. Clinical evaluation, hematological and biochemical parameters, and serum levels of cytokines were measured by conventional methods. The parasite burden in the bone marrow was measured by quantitative real-time PCR, and the transmissibility of parasites to sand flies was assessed by xenodiagnosis. Clinical, biochemical, and hematological parameters of vaccinated infected dogs were mostly normal. Vi dogs developed mild disease with low clinical scores. Serum levels of IL-10 were higher in Vi dogs, and a strong correlation was observed in IL-4 levels and the A/G ratio in Vi dogs. These results suggest a role of TH2 response in Vi dogs, although more data is needed to better understand the disease in vaccinated dogs.

## 1. Introduction

Zoonotic visceral leishmaniasis (VL) is a disease caused by the protozoa *Leishmania infantum* and is transmitted to mammalian hosts by the bites of female sand flies. Dogs are the most significant domestic reservoir responsible for its maintenance in endemic foci and are thus one of the strategic targets for disease control [1–3]. The phlebotomine sand fly *Lutzomyia longipalpis* is the main vector of disease in Brazil [4]. While blood feeding on a vertebrate host, females ingest amastigote-infected macrophages in the tissue. Once in the insect gut, amastigotes develop into promastigotes and, a few days later, into the infective forms known as metacyclic promastigotes. During their next blood meal, the female sand flies regurgitate the metacyclic promastigotes into the host's skin, thus completing the cycle of the transmission [4, 5]. Once inside the host, the parasites replicate inside macrophages and are disseminated throughout the body, infecting various tissues including the liver, spleen, and bone marrow [6].

Control measures focused on dogs include the use of insecticide collars and vaccination [7]. The Leishmune™ vaccine was the first commercial vaccine against canine leishmaniasis to be approved and used in Brazil. It is composed of the fucose-mannose ligand fraction purified from the promastigote *Leishmania donovani* membrane, along with *Quillaja saponaria* saponin as the adjuvant [8–10]. Leishmune™ was in use for at least ten years, but its sale is now suspended in Brazil as the phase III study was considered insufficient by the Ministry of Agriculture, Livestock, and Food Supply to maintain its license [11]. Additionally, several reports have attempted to demonstrate its efficacy either as a vaccine or as an immunotherapy agent [12, 13].

Clinical signs of canine leishmaniasis that are commonly described include dermatological disorders, lymph node enlargement, and a variable degree of weight loss [14]. However, dogs with no clinical signs, usually called asymptomatic, are prevalent even in experimental studies [15]. Thus, defining the clinical stage of infected dogs is a challenging task. To overcome this problem, the combined use of clinical scores and routine biomarkers such as red and white blood cell counts and clinical biochemistry has been encouraged by several authors to determine the clinical status of individuals [14, 16].

Hematological abnormalities are frequent in diseased dogs. Anemia is observed in 40 to 90% of infected animals [17, 18], being an important marker of disease severity [19]. Abnormal blood counts are positively correlated with the severity of clinical signs and with parasite density in the bone marrow [20, 21]. An increase in the myeloid : erythroid (M : E) ratio also correlates with general bone marrow dysplasia in infected animals [22], while biomarkers specific to hepatic function and to renal function in particular are variable depending on the stage of the disease. Dogs with VL often exhibit hyperproteinemia, characterized by hyperglobulinemia and hypoalbuminemia. Consequently, infected dogs present with albumin/globulin (A/G) ratios below the normal range [14].

Although some hematological and biochemical abnormalities are frequently found in infected dogs, there are no definitive standards to help with clinical staging. Biomarkers that are consistently stable during disease and that correlate with the clinical stage would be a remarkable tool. The levels of some cytokines produced during the disease are among the parameters that could be used to assess the general clinical status of dogs with leishmaniasis, but this is not the usual approach. The production of cytokines in certain tissues may provide some information about the immunopathological processes occurring within them. Several studies have assessed the immunopathological status of dogs by measuring the levels of cytokines produced by stimulated peripheral blood mononuclear cells *in vitro* [23–25]. Furthermore, the direct measurement of cytokines in serum is an approach that may provide some information about the ongoing systemic immunopathological processes; this may correlate with the clinical status and the parasite burden in a given animal [26] and is easier to perform than stimulating peripheral blood mononuclear cells *in vitro*.

Herein, we have described the serum levels of cytokines, clinical score, parasite load, and the reservoir potential of vaccinated noninfected dogs and naturally infected dogs that were previously vaccinated with Leishmune™. We have also evaluated the putative correlations between analytes and clinical parameters in vaccinated infected dogs.

## 2. Materials and Methods

### 2.1. Ethics Statement

All dogs used in this study were handled in accordance with animal practice as defined by the Internal Ethics Committee on Animal Experimentation (CEUA) of Universidade Federal de Minas Gerais, Belo Horizonte, Brazil (Protocol 32/2011). This protocol follows the guidelines of CONCEA/MCT, the animal ethics committee of Brazil. Before participating, all owners signed an informed consent form.

### 2.2. Animals

Domiciled dogs (*n* = 34) of different breeds and sexes living in the metropolitan region of Belo Horizonte, a VL-endemic area, were included in this study. Dogs were aged from one to five years and had been vaccinated with Leishmune™. The owners of all dogs that came to the hospital for revaccination (annual booster) with Leishmune™ who had a complete and regular history of vaccination were invited to join the study. Vaccination with Leishmune™ was performed according to the manufacturer's recommendations: dogs were first serologically tested for leishmaniasis, and after confirmation of a negative result, an initial three-dose schedule at 21-day intervals was administered, followed by at least an annual booster one year after the first dose [10]. All vaccinated dogs included in this study (*n* = 34) had received at least one annual booster and were sampled up to one year after their last annual booster. Vaccinated dogs were divided into two groups: vaccinated infected (*n* = 21, Vi) and vaccinated noninfected (*n* = 13, Vn) dogs.

Evidence of infection was assessed in the groups by xenodiagnosis, cytology, and quantitative polymerase chain reaction (qPCR) of the bone marrow. Dogs with clinical or laboratory evidence of concurrent disease were not included in this study. All dogs lived in the metropolitan region of Belo Horizonte, according to the owner's statements from anamnesis.

### 2.3. Disease Staging

Vaccinated infected dogs were categorized according to the disease stage, following a previously established protocol with some adaptations [27]. Briefly, stage I dogs have discrete signs of disease and no laboratory abnormalities, stage II dogs have moderate clinical signs and laboratory abnormalities, stage III dogs have severe clinical signs associated with vasculitis and evidence of renal injury, and stage IV dogs have severe clinical signs and chronic renal disease. Dogs with no clinical signs or laboratory abnormalities were assigned a clinical score of 0.

### 2.4. Sample Collection

Blood samples were collected from each animal after clinical examination, distributed equally into tubes with and without ethylenediaminetetraacetic acid (EDTA). Bone marrow sampling was performed under sedation with intramuscular injections of 0.05 mg/kg acepromazine (0.2%; Acepran, Univet, Louveira, SP, Brazil) and 0.1 mL/kg fentanyl citrate (0.05 mg/mL; Fentanest, Cristalia, Belo Horizonte, MG, Brazil). Samples from the *manubrium sterni* were collected under local anesthesia with 2% lidocaine (Hipolabor, Belo Horizonte, MG, Brazil). Glass slide smears were prepared with an aliquot immediately after collection, and the remaining sample was stored at -20°C for qPCR.

### 2.5. Blood Count and Serum Biochemistry

The whole blood collected in EDTA was processed in an automated hematology analyzer (Abacus Junior Vet, Diatron® Group, Budapest, Hungary) to obtain total leukocyte (WBC), erythrocyte (RBC), hemoglobin, and platelet counts. Packed cell volume (PCV) was evaluated by centrifugation of whole blood for 10 min using a microcapillary centrifuge (Microline microcentrifuge, Laborline, Barueri, SP, Brazil). Other red blood cell indices including mean corpuscular volume (MCV) and hemoglobin concentration (MCHC) were derived from the PCV, hemoglobin, and erythrocyte counts.

One blood smear per sample was stained with a routine hematologic (Romanowsky type) stain and evaluated by light microscopy (Olympus, CH 30, Tokyo, Japan) under an oil immersion objective (1000x) for differential leukocyte counts, as well as morphological evaluation of erythrocytes, leukocytes, and platelets.

The serum was separated by centrifugation of whole blood at 3000 rpm for 10 min and transferred to Eppendorf-type vials for automated analysis (Cobas Mira®, Roche, Basel, Switzerland). Biochemical parameters were evaluated, including blood urea nitrogen, creatinine, aspartate aminotransferase (AST), alanine aminotransferase (ALT), total protein, albumin, and globulin concentrations, as well as the A/G ratio. Each component was evaluated using a specific kit (SynerMed, Monterey Park, California, USA) according to the manufacturer's instructions.

Bone marrow smears were stained with a Romanowsky-type stain and subjected to cytological evaluation of the bone marrow smears and direct parasitological examination. The smears were examined under 10x magnification for the presence of particles, the percentage of cells, estimative iron concentration, and megakaryocyte counts. Under an oil immersion objective (1000x), 500 nucleated cells were enumerated to derive the M : E ratio and to verify the cellular distribution. Direct parasitological evaluation was initially performed on three slides per sample, with evaluation of additional slides as required for the identification of the parasite. Parasite density (mild, moderate, or severe) was determined according to the number of parasites per field under the oil immersion objective [28].

### 2.6. Quantitative PCR: Extraction and Amplification of DNA

*Leishmania* DNA was extracted according to a previously described protocol [29] and evaluated by qPCR using TaqMan probes followed by the construction of a standard qPCR curve for parasites of the genus *Leishmania* [30]. Customized primers (Applied Biosystems, Belo Horizonte, MG, Brazil) were used to amplify a conserved region in the *Leishmania* kinetoplast DNA minicircle [31]. The number of copies of *Leishmania* DNA/mL of bone marrow material was determined, and the results were plotted on the calibration standard curve.

### 2.7. Serum Cytokines

Serum samples were stored at -80°C for later analysis. Serum cytokines were measured by the Luminex bead-based multiplex assay for canine cytokines, following the manufacturer's instructions (Milliplex Canine Cytokine, Merck Millipore).

### 2.8. Xenodiagnosis

Dogs were exposed to approximately forty laboratory-raised 55^th^ generation *L. longipalpis* females. The protocol used for the feeding of insects on dogs has been described elsewhere [17]. Subsequently, the insects were kept in Center for Disease Control minitraps for seven days and fed a 10% sucrose solution. Seven days after blood feeding, the sand flies were dissected on a drop of 0.9% saline under a stereoscope (Labmex, PZO, Monterey, Mexico). Using a needle attached to an epoxy rod, the gut was removed through the posterior portion of the abdomen and covered with a coverslip. The guts of *L. longipalpis* females were examined for the presence of promastigotes under light microscopy (400x). Finally, the numbers of noninfected and infected sand flies per total number of dissected sand flies were determined for each dog in the study.

### 2.9. Statistical Analysis

Gaussian distribution was inferred using the Shapiro-Wilk normality test. Normally distributed data were evaluated by unpaired Student's *t*-test, while nonnormally distributed data were evaluated by the Mann-Whitney test. Correlations between parameters were established using the Spearman method, and associations were assessed by Fisher's exact test, both with 95% confidence intervals. Correlations were interpreted according to values of the correlation coefficient, as follows: negligible (0.0 to 0.2), weak (0.2 to 0.4), moderate (0.4 to 0.6), strong (0.6 to 0.8), and very strong (0.8 to 1.0). Statistical analyses were performed with GraphPad Prism 5.0 (GraphPad Software, Inc., San Diego, CA).

## 3. Results

### 3.1. Staging and Clinical Signs of Canine VL

All dogs were tested by different methods to determine the evidence of infection with *L. infantum* parasites.  Table 1 shows the number of dogs among the vaccinated infected group in each test. Out of the 34 vaccinated dogs, 13 (38.2%) had no evidence of infection in any test and were therefore allocated to the Vn group. Of the 21 dogs in the vaccinated infected group (Vi): 13/21 (61.9%), 12/20 (60.0%), and 1/18 (5.5%) tested positive by xenodiagnosis, bone marrow qPCR, and bone marrow cytology, respectively. Only one dog was positive in all tests, while 3 were positive in both xenodiagnosis and BM-qPCR. Seven dogs were positive only in BM-qPCR and 6 only in the xenodiagnoses (Table 1). Bone marrow cytology was not performed in three dogs from the Vi group due to rapid coagulation of the sample during collection, and xenodiagnosis was not performed in one dog. Xenodiagnosis was performed in only 10 dogs of the Vn group, due to owner refusal.

Among Vi dogs, 8 (38.1%) had no clinical signs compatible with leishmaniasis, while 13 (61.9%) had clinical signs compatible with lower clinical scores (Table 2). A high percentage of Vi dogs (17/21, 81%) had mild signs of clinical disease, mainly consisting of scarce dermatologic lesions, lymph node enlargement, and/or splenomegaly. No clinical signs were observed in Vn dogs. Importantly, among Vi dogs, nine (40.9%) were positive on xenodiagnosis, but negative on bone marrow qPCR.

### 3.2. Hematological and Biochemical Parameters

No significant difference in red blood cell counts was observed between Vi and Vn dogs. Moreover, no differences were observed in the M : E ratio between Vi and Vn dogs. Anemia (defined as PCV below the reference value) was seen in 4/21 (19.0%) in the Vi group (Fig. S1). Serum levels of blood urea nitrogen, creatinine, AST, and ALT were also similar across all groups (Fig. S2).

### 3.3. Reservoir Potential

Reservoir potential is defined here as the capacity of a host to infect sand flies and is measured as the percentage of infected sand flies per dog. Xenodiagnosis was successfully performed in 30 dogs, and among those infected with *L. infantum*, the percentage of infected female sand flies ranged from 0 to 32%. Among the 13 Vi dogs with positive xenodiagnoses, 4 had clinical score 0, thus considered asymptomatic reservoir. No significant correlation was observed between clinical scores and reservoir potential. Also, there was no correlation between the rate of infected sand flies and the parasite load in the BM when considering the Vi group. Interestingly, 9 Vi dogs were positive in xenodiagnosis while negative in BM-qPCR. Of the ten dogs in the Vn group, none were positive by xenodiagnosis.

### 3.4. Serum Cytokine Profile

**Figure 1:**
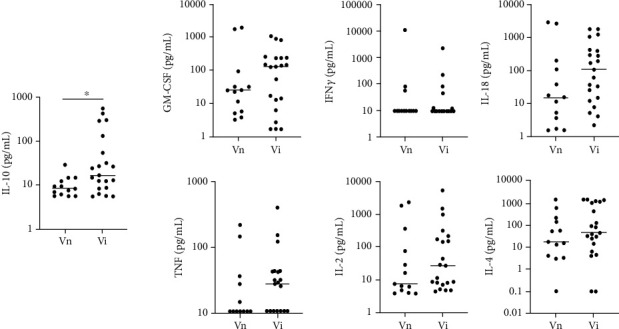
Serum levels of cytokines in dogs vaccinated with Leishmune™ infected or not by *Leishmania infantum*. The *Y*-axis is transformed as log_10_. Dots represent the individual values of each dog. Lines represent the median of each group. Vn: vaccinated not infected dogs; Vi: vaccinated infected dogs. Mann-Whitney *U* test. ^∗^*p* < 0.05.

Levels of IFN-*γ* were undetectable in most samples (below 9.4 pg/mL); however, 12 dogs from the vaccinated groups (Vn and Vi) had IFN-*γ* levels ranging from 12.0 pg/mL to 10.9 ng/mL. Among tested cytokines, only IL-10 was significantly higher in Vi dogs than in Vn (Figure 1).

**Figure 2:**
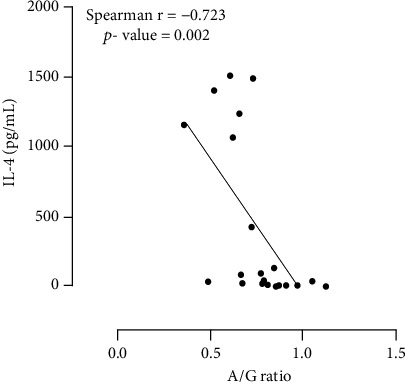
Correlation between IL-4 and A/G ratio among Vi dogs. Dots represent individual values of each dog. Lines represent the linear regression model of presented data. Statistical significance is plotted as Spearman's rank correlation coefficient (Spearman *r*) and *p* value.

A strong positive correlation was found between serum levels of cytokines TNF-*α*, IL-18, GM-CSF, and IL-2 (*r* > 0.6; Table 3) when compared in pairs. A very strong positive correlation was found between GM-CSF and IL-18 (*r* > 0.8; Table 3). A strong correlation was observed between serum levels of IL-4 and A/G ratio in the Vi group (*r* = −0.723, CI = −0.883 to -0.412, *p* = 0.0002, Figure 2). No correlation was observed between serum levels of cytokines and any other variables evaluated.

## 4. Discussion

In this study, we assessed the clinical data, parasitological parameters, and the serum cytokine profile of vaccinated dogs with the first-generation vaccine against leishmaniasis developed in Brazil. This was a transversal descriptive study with relevant data relating to naturally infected dogs that were previously vaccinated against canine VL. We focused on comparing the data available between vaccinated dogs that were infected with those that were not (Vi and Vn, respectively). Data from vaccinated dogs that become infected are often neglected. Here, we present data that represent the dogs' parameters at some point during infection. While this may not predict the outcome of canine VL, our results will hopefully inform future studies to shed further light on this disease.

Sixty-two percent of the vaccinated dogs (21/34) were positive on at least one of the parasitological tests performed in the study. Dermatologic lesions and lymphadenopathy were the most common clinical signs observed in this study and are in accordance with previous findings [16]. Vi dogs had mild clinical signs which agreed with previous findings that report a lower incidence of clinical signs in FML-vaccinated dogs that became infected (5 to 8%) compared to unvaccinated dogs (25 to 33%) [32, 33]. This is an important characteristic of vaccines used against diseases that result in severe infections, such as canine VL. It has been shown that vaccination with Leishmune™ interferes with the development of *L. infantum* in sand flies due to impairment of promastigote adhesion in the sand fly midgut by anti-FML antibodies [13], which is of importance for public health. The transmission blocking activity is an interesting feature of a vaccine against leishmaniasis, which has also been shown by others [34].

Anemia is the most common hematological disorder in dogs with leishmaniasis [14, 35, 36]. Vi dogs commonly displayed neither anemia nor medullary dyspoiesis. Although reported by others [18, 35, 36], alterations in the absolute counts of different leukocytes were not present between the groups in our study. In the present study, dogs within the Vi group did not display a decreased A/G ratio compared to Vn dogs. Increased levels of serum proteins and globulins are commonly detected in dogs with leishmaniasis, while levels of albumin and the A/G ratio are frequently decreased [36, 37]. The A/G ratio has been extensively used by veterinary practitioners to monitor the clinical course of the disease [20, 38, 39]. An increase in serum levels of BUN and creatinine only occurs several months after infection, and chronic kidney disease is a frequent finding in dogs chronically infected with *Leishmania*, representing the final stage of the disease [14]. Other abnormalities on laboratory tests are highly variable in canine leishmaniasis and may be present due to concurrent diseases and conditions [36]. Interestingly, nine dogs, all belonging to the Vi group, were positive on xenodiagnosis, but negative on BM-qPCR. This is relevant to public health since all of these dogs were asymptomatic or were assigned the lowest clinical score. However, this is a transversal study, and since dogs were not followed up, we cannot assume the progression of the disease or their reservoir potential in chronic disease.

Taken together, these results suggest the potential role of the vaccine as a means of protecting dogs from being infected or developing severe disease, which is corroborated by others [8, 17]. This potential has been explored as an immunotherapeutic option with a certain degree of success and should be considered in future studies [12, 40]. However, considering that some vaccinated dogs may become infected, it is critical to determine whether clinical scores remain low during chronic infection and if chronically infected vaccinated dogs are a poor source of infection to sand flies. Also, until now, there is no solid scientific evidence of reduced transmission of *Leishmania infantum* from infected vaccinated dogs to sand flies to a level that would significantly reduce the risk of infection of visceral leishmaniasis in humans [41].

The strong correlations between levels of TNF-*α*, IL-18, GM-CSF, and IL-2 might be explained by the concomitant expression of these cytokines during inflammation. While TNF-*α* and IL-18 are mainly produced by macrophages, GM-CSF is produced by several cell types including macrophages, which is the predominant cell type in lesions caused by VL [42]. While TNF-*α* and IL-18 are cytokines directly involved in effector mechanisms within the inflammatory milieu, GM-CSF and IL-2 are involved in cell proliferation and thus indirectly involved in inflammation [43]. TNF-*α* and IL-18 are frequently associated with resistance against *Leishmania* due to their contribution to the resistance phenotype in murine models [44].

The levels of cytokines are commonly evaluated by stimulation of peripheral blood mononuclear cells with *Leishmania* antigens. This approach allows us to detect an antigen-specific immune response against *Leishmania* in an individual and is considered the gold standard for evaluation of the immune response in these animals [45]. Direct measurement of cytokine levels in serum is an approach that has been used extensively and is evolving to deliver more accurate results [46–49]. Whether the cytokine levels in serum accurately portray the systemic immune response in an individual or not is yet to be determined. However, it is an interesting and practical tool that is worthy of further study.

The levels of cytokines might be altered depending on the time point evaluated, varying greatly during disease. Additionally, they might be strongly determined by the organ samples, methodological approach, and animals used in the study. Our approach in measuring cytokines directly from serum samples is a practical and straightforward strategy to, in effect, assess a general immunologic index of a patient. New studies are needed to verify the value of this approach and the use of cytokines as potential biomarkers for disease progression or even for the assessment of treatment efficacy.

Data on serum levels of cytokines during VL are mostly absent, but one study reported no significant differences in levels of TNF-*α* between infected and uninfected dogs [50]. This result was corroborated by another approach [25, 51]. The levels of IL-10 were higher in Vi dogs compared to Vn dogs (Figure 1). Levels of IL-10 were also found elevated in infected dogs by different approaches compared to uninfected dogs [25, 51]. IL-10 has been associated with the suppression of TH1 cytokines, skewing the immune response towards a TH2 immune response, especially in cases of human visceral leishmaniasis [47].

It is worth noting that the levels of cytokines vary immensely between individuals, with some individuals having extremely high levels of some cytokines. This finding suggests the presence of a mixed-type immune profile, as infected dogs displayed higher levels of cytokines with no clear correlation with the polarized immune response observed in murine models [44, 45]. The lack of a polarized TH1/TH2 immune response in canine leishmaniasis has been suggested by others [24, 41, 47] as has a compartmented immune response with different cytokine profiles in certain tissues [52]. In both cases, serum levels of cytokines may represent a systemic view of the immune responses occurring in the tissues.

We found a strong correlation between the levels of IL-4 in the serum and the albumin/globulin ratio in Vi dogs (Figure 2). A low A/G ratio is commonly observed in infected dogs as a result of the increase of gamma-globulin due to polyclonal activation of B cells and is commonly related to a worsening in the disease [20, 36–39]. In murine models, the production of IL-4 is increased in susceptible mice, but not in resistant mice [44]. However, in dogs, levels of IL-4 are not directly related to susceptibility, as dogs commonly display a mixed TH1/TH2 immune response [52]. Others have shown that the number of T cells expressing IL-4 in dogs vaccinated with Leishmune™ is comparable to those in uninfected dogs [51]. Levels of IL-4 are increased in the skin and bone marrow of some infected dogs [53, 54] and in the serum of infected humans [55]. Taken together, our results suggest a role of the TH2 response in Vi dogs with a possible correlation with disease development. Again, this is a transversal study, and since dogs were not followed up, we cannot make confident assumptions on how the disease developed in Vi dogs. More data is needed to verify whether Vi dogs develop mild disease compared to nonvaccinated dogs and the role of their immunity in the disease outcome.

In conclusion, we observed that although vaccinated dogs become infected, they may display discreet clinical signs and still be infective to sand flies. However, vaccination might also impact reservoir potential by limiting the percentage of sand flies that are infected after blood feeding. The value of serum cytokine measurements alone is limited, but in association with other parameters, they might be useful for evaluating disease progression in some individuals.

## Figures and Tables

**Table 1:** Distribution of subjects in Vi group by infection status for each diagnostic method.

Test	Result	#	%
Xenodiagnosis	Positive	13	61.9%
	Negative	7	33.3%
BM-qPCR	Positive	12	60%
	Negative	8	40%
BM-cytology	Positive	1	5.5%
	Negative	17	94.4%

BM: bone marrow.

**Table 2:** Distribution of subjects between groups and corresponding clinical score.

	Infected (Vi)		Not infected (Vn)	
Clinical score	#	%	#	%
0	8	38.1%	13	100%
I	9	42.9%	0	0%
II	4	19.0%	0	0%
III	0	0%	0	0%
IV	0	0%	0	0%
Total	21	100%	13	100%

**Table 3:** Correlations between serum cytokine levels.

		*p* value						
		GM-CSF	IFN-*γ*	IL-2	IL-10	IL-18	TNF-*α*	IL-4
Spearman *r*	GM-CSF		0.2501	<0.0001	0.8915	<0.0001	<0.0001	0.5471
	IFN-*γ*	-0.2167		0.2731	0.1575	0.4634	0.5105	0.8808
	IL-2	0.7311	-0.2067		0.9547	<0.0001	<0.0001	0.8761
	IL-10	0.0260	0.2647	0.0108		0.2918	0.2980	0.1250
	IL-18	0.8361	-0.1391	0.6850	0.1990		<0.0001	0.7828
	TNF-*α*	0.7588	0.1250	0.6770	-0.1965	0.7975		0.6376
	IL-4	0.1144	0.0286	0.0297	0.2863	0.0525	0.0896	

## Data Availability

Complementary data can be obtained from the authors by request.

## Supplementary Materials

Supplementary Materials Fig. S1: hematological features of vaccinated infected (Vi) and vaccinated not infected (Vn) dogs. Fig. S2: serum biochemistry features of vaccinated infected (Vi) and vaccinated not infected (Vn) dogs.

## References

[1] Costa C. H. N. (2011). How effective is dog culling in controlling zoonotic visceral leishmaniasis? A critical evaluation of the science, politics and ethics behind this public health policy. *Revista da Sociedade Brasileira de Medicina Tropical*.

[2] Costa C. H. N. (2008). Characterization and speculations on the urbanization of visceral leishmaniasis in Brazil. *Cadernos de Saúde Pública*.

[3] Moreno J., Alvar J. (2002). Canine leishmaniasis: epidemiological risk and the experimental model. *Trends in Parasitology*.

[4] Soares R. P. P., Turco S. J. (2003). Lutzomyia longipalpis (Diptera: Psychodidae: Phlebotominae): a review. *Anais da Academia Brasileira de Ciências*.

[5] Kamhawi S. (2006). Phlebotomine sand flies and *Leishmania* parasites: friends or foes?. *Trends in Parasitology*.

[6] Miró G., Cardoso L., Pennisi M. G., Oliva G., Baneth G. (2008). Canine leishmaniosis - new concepts and insights on an expanding zoonosis: part two. *Trends in Parasitology*.

[7] Sevá A. P., Ovallos F. G., Amaku M. (2016). Canine-based strategies for prevention and control of visceral leishmaniasis in Brazil. *PLoS One*.

[8] Nogueira F. S., Moreira M. A. B., Borja-Cabrera G. P. (2005). Leishmune^®^ vaccine blocks the transmission of canine visceral leishmaniasis:. *Vaccine*.

[9] Borja-Cabrera G. P., Santos F. B., Picillo E., Gravino A. E., Manna L., Palatnik-de-Sousa C. B. (2009). Nucleoside hydrolase DNA vaccine against canine visceral leishmaniasis. *Procedia in Vaccinology*.

[10] Palatnik-de-Sousa C. B., Silva-Antunes I., Morgado A. D. A., Menz I., Palatnik M., Lavor C. (2009). Decrease of the incidence of human and canine visceral leishmaniasis after dog vaccination with Leishmune^®^ in Brazilian endemic areas. *Vaccine*.

[11] B. Ministry of Agriculture, Livestock and Food Supply nota técnica n.038/2014/DFIP/SDA. http://www.agricultura.gov.br/assuntos/insumos-agropecuarios/insumos-pecuarios/produtos-veterinarios/arquivos-comunicacoes-e-instrucoes-tecnicas/nota-tecnica-dfip-38-14-leishmune.pdf.

[12] Borja-Cabrera G. P., Santos F. N., Santos F. B. (2010). Immunotherapy with the saponin enriched-Leishmune^®^ vaccine *versus* immunochemotherapy in dogs with natural canine visceral leishmaniasis. *Vaccine*.

[13] Saraiva E. M., Barbosa A. D. F., Santos F. N. (2006). The FML-vaccine (Leishmune®) against canine visceral leishmaniasis: a transmission blocking vaccine. *Vaccine*.

[14] Solano-Gallego L., Miró G., Koutinas A. (2011). LeishVet guidelines for the practical management of canine leishmaniosis. *Parasites Vectors*.

[15] Killick-Kendrick R., Killick-Kendrick M., Pinelli E. (1994). A laboratory model of canine leishmaniasis: the inoculation of dogs withLeishmania infantumpromastigotes from midguts of experimentally infected phlebotomine sandflies. *Parasite*.

[16] Meléndez-Lazo A., Ordeix L., Planellas M., Pastor J., Solano-Gallego L. (2018). Clinicopathological findings in sick dogs naturally infected with *Leishmania infantum* : Comparison of five different clinical classification systems. *Research in Veterinary Science*.

[17] De Amorim I. F. G., Freitas E., Alves C. F. (2010). Humoral immunological profile and parasitological statuses of Leishmune^®^ vaccinated and visceral leishmaniasis infected dogs from an endemic area. *Veterinary Parasitology*.

[18] Nicolato R. D. C., De Abreu R. T., Roatt B. M. (2013). Clinical forms of canine visceral leishmaniasis in naturally Leishmania infantum-infected dogs and related myelogram and hemogram changes. *PLoS One*.

[19] Travi B. L., Osorio E. Y., Saldarriaga O. A. (2009). Clinical, parasitologic, and immunologic evolution in dogs experimentally infected with sand fly-derived Leishmania chagasi promastigotes. *The American Journal of Tropical Medicine and Hygiene*.

[20] Reis A. B., Martins-Filho O. A., Teixeira-Carvalho A. (2006). Parasite density and impaired biochemical/hematological status are associated with severe clinical aspects of canine visceral leishmaniasis. *Research in Veterinary Science*.

[21] Trópia de Abreu R., Carvalho M. D. G., Carneiro C. M. (2011). Influence of clinical status and parasite load on erythropoiesis and leucopoiesis in dogs naturally infected with Leishmania (Leishmania) chagasi. *PLoS One*.

[22] Manzillo V. F., Restucci B., Pagano A., Gradoni L., Oliva G. (2006). Pathological changes in the bone marrow of dogs with leishmaniosis. *The Veterinary Record*.

[23] Aslan H., Oliveira F., Meneses C. (2016). New insights into the transmissibility ofLeishmania infantumFrom dogs to sand flies: experimental vector-transmission reveals persistent parasite depots at bite sites. *The Journal of Infectious Diseases*.

[24] Panaro M. A., Brandonisio O., Cianciulli A. (2009). Cytokine expression in dogs with naturalLeishmania infantuminfection. *Parasitology*.

[25] Chamizo C., Moreno J., Alvar J. (2005). Semi-quantitative analysis of cytokine expression in asymptomatic canine leishmaniasis. *Veterinary Immunology and Immunopathology*.

[26] Tarrant J. M. (2010). Blood cytokines as biomarkers of in vivo toxicity in preclinical safety assessment: considerations for their use. *Toxicological Sciences*.

[27] Solano-Gallego L., Koutinas A., Miró G. (2009). Directions for the diagnosis, clinical staging, treatment and prevention of canine leishmaniosis. *Veterinary Parasitology*.

[28] Nunes C. M., Pires M. M., da Silva K. M., Assis F. D., Filho J. G., Perri S. H. V. (2010). Relationship between dog culling and incidence of human visceral leishmaniasis in an endemic area. *Veterinary Parasitology*.

[29] Barea J. A., Pardini M. I. M. C., Gushiken T. (2004). Extração de DNA de materiais de arquivo e fontes escassas para utilização em reação de polimerização em cadeia (PCR). *Revista Brasileira de Hematologia e Hemoterapia*.

[30] van der Meide W., Guerra J., Schoone G. (2008). Comparison between quantitative nucleic acid sequence-based amplification, real-time reverse transcriptase PCR, and real-time PCR for quantification ofLeishmaniaParasites. *Journal of Clinical Microbiology*.

[31] Rodgers M. R., Popper S. J., Wirth D. F. (1990). Amplification of kinetoplast DNA as a tool in the detection and diagnosis of *Leishmania*. *Experimental Parasitology*.

[32] Borja-Cabrera G. P., Correia Pontes N. N., Da Silva V. O. (2002). Long lasting protection against canine kala-azar using the FML-QuilA saponin vaccine in an endemic area of Brazil (Sao Gonçalo do Amarante, RN). *Vaccine*.

[33] Da Silva V. O., Borja-Cabrera G. P., Correia Pontes N. N. (2000). A phase III trial of efficacy of the FML-vaccine against canine kala-azar in an endemic area of Brazil (Sao Gonçalo do Amaranto, RN). *Vaccine*.

[34] Bongiorno G., Paparcone R., Manzillo V. F., Oliva G., Cuisinier A. M., Gradoni L. (2013). Vaccination with LiESP/QA-21 (CaniLeish^®^) reduces the intensity of infection in *Phlebotomus perniciosus* fed on *Leishmania infantum* infected dogs --A preliminary xenodiagnosis study. *Veterinary Parasitology*.

[35] De Tommasi A. S., Otranto D., Furlanello T. (2014). Evaluation of blood and bone marrow in selected canine vector-borne diseases. *Parasites & Vectors*.

[36] Paltrinieri S., Gradoni L., Roura X., Zatelli A., Zini E. (2016). Laboratory tests for diagnosing and monitoring canine leishmaniasis. *Veterinary Clinical Pathology*.

[37] Proverbio D., Spada E., Bagnagatti De Giorgi G., Perego R., Valena E. (2014). Relationship between Leishmania IFAT titer and clinicopathological manifestations (clinical score) in dogs. *BioMed Research International*.

[38] Giunchetti R. C., Mayrink W., Carneiro C. M. (2008). Histopathological and immunohistochemical investigations of the hepatic compartment associated with parasitism and serum biochemical changes in canine visceral leishmaniasis. *Research in Veterinary Science*.

[39] Ribeiro R. R., Silva S. M. ., Fulgêncio G. . O., Michalick M. S. M., Frézard F. J. G. (2013). Relationship between clinical and pathological signs and severity of canine leishmaniasis. *Revista Brasileira de Parasitologia Veterinária*.

[40] Santos F. N., Borja-Cabrera G. P., Miyashiro L. M. (2007). Immunotherapy against experimental canine visceral leishmaniasis with the saponin enriched-Leishmune^®^ vaccine. *Vaccine*.

[41] Dantas-Torres F., Nogueira F. . S., Menz I. (2020). Vaccination against canine leishmaniasis in Brazil. *International journal for parasitology*.

[42] Koutinas A. F., Koutinas C. K. (2014). Pathologic mechanisms underlying the clinical findings in canine leishmaniosis due *toLeishmaniainfantum/chagasi*. *Veterinary Pathology*.

[43] Feghali C. A., Wright T. M. (1997). Cytokines in acute and chronic inflammation. *Frontiers in Bioscience*.

[44] Sacks D., Noben-Trauth N. (2002). The immunology of susceptibility and resistance to *Leishmania major* in mice. *Nature Reviews. Immunology*.

[45] Hosein S., Blake D. P., Solano-Gallego L. (2017). Insights on adaptive and innate immunity in canine leishmaniosis. *Parasitology*.

[46] Karlsson I., Hagman R., Johannisson A., Wang L., Karlstam E., Wernersson S. (2012). Cytokines as immunological markers for systemic inflammation in dogs with pyometra. *Reproduction in Domestic Animals*.

[47] Fransson B. A., Lagerstedt A. S., Bergstrom A. (2007). C-reactive protein, tumor necrosis factor *α*, and interleukin-6 in dogs with pyometra and SIRS. *Journal of Veterinary Emergency and Critical Care*.

[48] Langhorn R., Oyama M. A., King L. G. (2013). Prognostic importance of myocardial injury in critically ill dogs with systemic inflammation. *Journal of Veterinary Internal Medicine*.

[49] Zois N. E., Moesgaard S. G., Kjelgaard-Hansen M. (2012). Circulating cytokine concentrations in dogs with different degrees of myxomatous mitral valve disease. *Veterinary Journal*.

[50] de Lima V. M. F., Peiro J. R., de Oliveira Vasconcelos R. (2007). IL-6 and TNF-*α* production during active canine visceral leishmaniasis. *Veterinary Immunology and Immunopathology*.

[51] Araújo M. S. S., de Andrade R. A., Sathler-Avelar R. (2009). T-cell-derived cytokines, nitric oxide production by peripheral blood monocytes and seric anti- *Leishmania (Leishmania) chagasi* IgG subclass patterns following immunization against canine visceral leishmaniasis using Leishvaccine and Leishmune ^®^. *Vaccine*.

[52] Reis A. B., Martins-Filho O. A., Teixeira-Carvalho A. (2009). Systemic and compartmentalized immune response in canine visceral leishmaniasis. *Veterinary Immunology and Immunopathology*.

[53] Quinnell R. J., Courtenay O., Shaw M. A. (2001). Tissue cytokine responses in canine visceral leishmaniasis. *The Journal of Infectious Diseases*.

[54] Brachelente C., Müller N., Doherr M. G., Sattler U., Welle M. (2005). Cutaneous leishmaniasis in naturally infected dogs is associated with a T helper-2-biased immune response. *Veterinary Pathology*.

[55] Cillari E., Vitale G., Arcoleo F. (1995). In vivo and in vitro cytokine profiles and mononuclear cell subsets in Sicilian patients with active visceral leishmaniasis. *Cytokine*.

