# Supplementary material for: FML/QuilA-Vaccinated Dogs Naturally Infected with Leishmania infantum: Serum Cytokines, Clinicopathological Profile, and Parasitological Parameters
Source: Biomed Res Int. 2021 Oct 5;2021:3192960. doi: 10.1155/2021/3192960 (PMC8510802; doi:10.1155/2021/3192960)
Supplement: Supplementary Materials — Fig. S1: hematological features of vaccinated infected (Vi) and vaccinated not infected (Vn) dogs. Fig. S2: serum biochemistry features of vaccinated infected (Vi) and vaccinated not infected (Vn) dogs. [file 3192960.f1.docx]

**
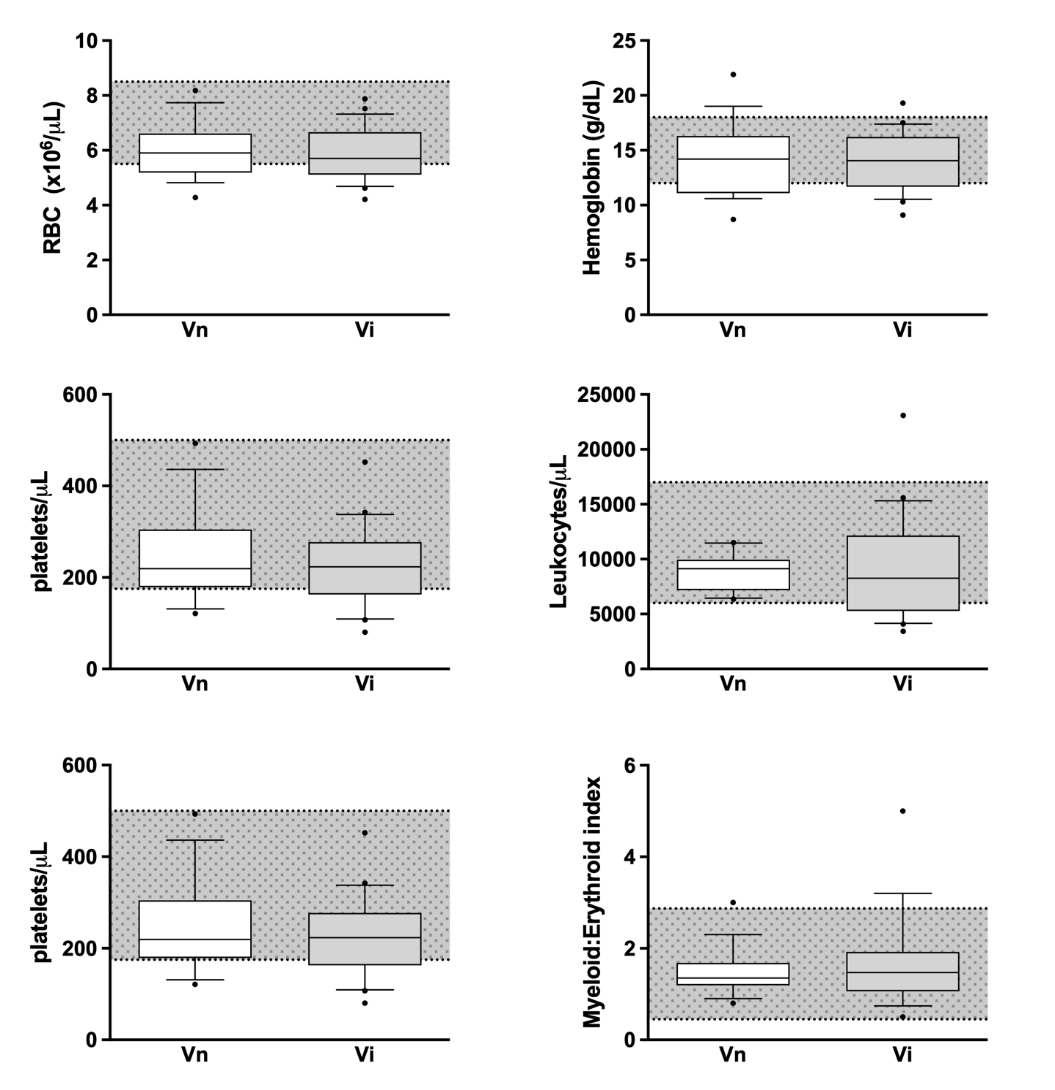
**

**Supplementary figure 1** - Hematological features of vaccinated infected (Vi) and vaccinated not infected (Vn) dogs vaccinated.


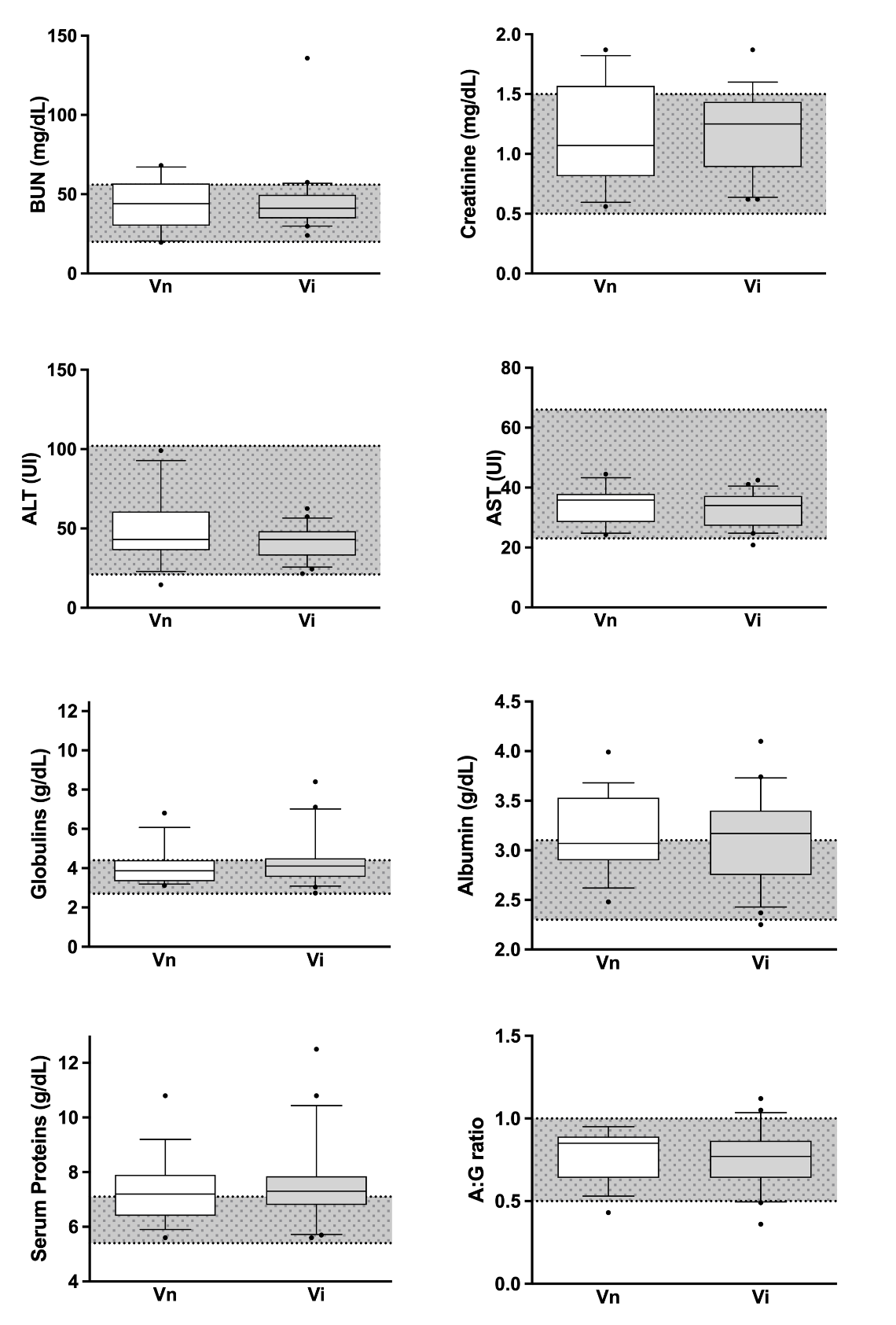


**Supplementary figure 2**- Serum biochemistry features of vaccinated infected (Vi) and vaccinated not infected (Vn) dogs vaccinated.
